# Supplementary material for: Volatile Profiles of Five Variants of Abeliophyllum distichum Flowers Using Headspace Solid-Phase Microextraction Gas Chromatography–Mass Spectrometry (HS-SPME-GC-MS) Analysis
Source: Plants (Basel). 2021 Jan 24;10(2):224. doi: 10.3390/plants10020224 (PMC7912044; doi:10.3390/plants10020224)
Supplement: Supplementary file 1 [file plants-10-00224-s001.pdf]

Supplementary Materials

Volatile profiles of five variants of *Abeliophyllum distichum* flowers using headspace-solid phase microextraction-gas chromatography–mass spectrometry (HS-SPME-GC-MS) analysis

Yeong-Geun Lee [1], Won-Sil Choi [2], Seung-Ok Yang [2], Jeon Hwang-Bo [1], Hyoung-Geun Kim [1], Minzhe Fang [1], Tae-Hoo Yi [1], Se Chan Kang [1], Youn-Hyung Lee [3] and Nam-In Baek [1,*]

[1] Graduate School of Biotechnology and Department of Oriental Medicine Biotechnology, Kyung Hee University, Yongin 17104, Republic of Korea

[2] National Instrumentation Center for Environmental Management, Seoul National University, Seoul 08826, Republic of Korea

[3] Department of Horticultural Biotechnology, Kyung Hee University, Yongin 17104, Republic of Korea

*Corresponding author

Phone: +82-31-201-2661; Fax: +82-31-204-8116

E-mail: nibaek@khu.ac.kr

Table Legends

Table S1. The detected volatile components identified from the flowers in five variants of *Abeliophyllum distichum* using HS-SPME-GC/MS analysis

Table S2. Classification and odor note of the detected volatile components identified from the flowers in five variants of *Abeliophyllum distichum* using HS-SPME-GC/MS analysis

Figure Legends

Figure S1. Representative total ion chromatograms (TIC) for raw floral tissue of five variants of *Abeliophyllum distichum*. (A) White miseon, (B) pink miseon, (C) ivory miseon, (D) blue miseon, (E) round miseon.

Figure S2. PCA score plot obtained from HS-SPME-GC/MS results on five variants of *Abeliophyllum distichum* flowers.

Figure S3. PLS-DA score plot obtained from HS-SPME-GC/MS results on five variants of *Abeliophyllum distichum* flowers.

Table S1. The detected volatile components identified from the flowers in five variants of *Abeliophyllum distichum* using HS-SPME-GC/MS analysis

No	Compound	RT[a]	RRT[b]	LRI[c]	LMV[d]	QI[e]	Mass fragment [*m*/*z*, (Relative intensity by comparison of biggest peak)][f]
1	isovaleraldehyde	11.50	0.352	885	85	**58**	41 (100), 58 (73), 57 (73), 44 (48), 43 (44), 39 (32), 71 (16), 42 (12)
2	ethanol	11.91	0.364	891	98	**45**	45 (100), 46 (27), 43 (17)
3	decane	15.26	0.467	1000	98	**57**	57 (100), 43 (93), 71 (76), 41 (36), 85 (31), 70 (29), 55 (19), 56 (19), 84 (17), 42 (13)
4	5-ethyl-2,2,3-trimethylheptane	16.82	0.515	1040	85	**57**	57 (100), 56 (43), 71 (40), 43 (30), 41 (21), 70 (19), 55 (13), 85 (11)
5	*n*-hexanal	17.67	0.541	1061	91	**57**	41 (100), 56 (96), 43 (87), 57 (87), 44 (79), 71 (50), 55 (30), 39 (30), 72 (28), 85 (25)
6	2-butyl-3-octanol	19.22	0.588	1101	92	**57**	43 (100), 69 (97), 70 (92), 57 (79), 55 (62), 71 (57), 41 (51), 83 (35), 84 (27), 111 (25)
7	*β*-myrcene	20.94	0.641	1146	88	**93**	93 (100), 41 (64), 69 (62), 91 (34), 77 (18), 79 (17), 39 (15), 67 (14), 92 (13)
8	isoamylalcohol	21.98	0.672	1174	91	**41**	41 (100), 55 (88), 57 (86), 70 (76), 56 (74), 42 (49), 43 (38), 39 (27), 45 (13)
9	limonene	22.62	0.692	1191	97	**93**	67 (100), 68 (88), 93 (84), 79 (53), 94 (37), 92 (35), 91 (32), 121 (28), 107 (26), 77 (26)
10	2-hexenal	22.91	0.701	1198	97	**41**	41 (100), 55 (93), 69 (88), 83 (74), 39 (63), 57 (59), 42 (56), 70 (27), 43 (27), 56 (24)
11	ocimene-1	23.54	0.720	1215	95	**93**	93 (100), 91 (57), 92 (42), 79 (39), 77 (37), 41 (18), 105 (17), 80 (15), 39 (15), 55 (12)
12	ocimene-2	24.21	0.741	1234	95	**93**	93 (100), 91 (72), 79 (52), 77 (40), 80 (36), 92 (29), 105 (23), 41 (22), 39 (16), 121 (16)
13	*n*-hexyl acetate	24.85	0.760	1251	93	**43**	43 (100), 56 (60), 61 (31), 55 (30), 69 (26), 41 (23), 42 (21), 84 (21), 73 (11)
14	isohexanol	25.95	0.794	1281	91	**56**	56 (100), 41 (62), 69 (51), 43 (45), 42 (36), 55 (24), 57 (21), 39 (15)
15	3-methyl-1-pentanol	26.43	0.809	1294	92	**56**	56 (100), 69 (74), 55 (68), 41 (52), 43 (50), 57 (26), 39 (15), 42 (15), 84 (13), 83 (12)
16	2-hexenyl acetate	26.92	0.823	1307	87	**67**	43 (100), 67 (61), 82 (37), 41 (35), 55 (31), 57 (27), 39 (22), 100 (21), 83 (17), 56 (14)
17	6-methyl-5-hepten-2-one	27.19	0.832	1315	85	**108**	43 (100), 41 (61), 69 (61), 55 (53), 108 (52), 111 (29), 83 (27), 71 (27), 67 (26), 93 (22)
18	1-hexanol	27.32	0.836	1319	97	**56**	56 (100), 55 (56), 43 (47), 41 (40), 69 (37), 42 (33), 39 (12)
19	*E*-3-eicosene	27.49	0.841	1324	86	**111**	69 (100), 43 (70), 57 (64), 55 (52), 83 (51), 85 (48), 41 (45), 84 (44), 111 (42), 71 (42)
20	2-methyl-1-decanol	27.80	0.850	1333	86	**85**	69 (100), 43 (82), 57 (71), 55 (55), 85 (52), 41 (47), 71 (46), 83 (45), 84 (43), 111 (42)
21	*Z*-3-hexenol	28.47	0.871	1352	89	**67**	67 (100), 41 (81), 82 (44), 55 (38), 39 (27), 69 (23), 42 (20), 57 (15), 54 (13), 53 (12)
22	allo-ocimene	28.60	0.875	1355	91	**121**	121 (100), 105 (56), 136 (42), 79 (38), 91 (36), 77 (28), 41 (27), 93 (24), 119 (13), 39 (13)
23	*p*-cymene	28.65	0.876	1357	91	**134**	119 (100), 91 (33), 134 (30), 121 (22), 105 (21), 57 (16), 41 (16), 77 (14), 71 (14), 43 (11)
24	2-hexenol	29.14	0.891	1371	88	**57**	57 (100), 41 (36), 82 (23), 67 (21), 39 (16), 43 (16), 44 (14), 55 (12), 71 (12), 56 (11)
25	nonaldehyde	29.39	0.899	1378	96	**41**	57 (100), 41 (84), 56 (61), 43 (54), 55 (50), 44 (46), 70 (45), 69 (36), 68 (35), 81 (35)
26	acetic acid	30.42	0.931	1408	94	**60**	43 (100), 45 (86), 60 (71), 42 (18), 83 (15), 55 (12), 41 (12), 57 (11), 97 (11)
27	1-octen-3-ol	30.61	0.936	1413	93	**57**	57 (100), 43 (25), 41 (23), 55 (23), 72 (16), 85 (12), 39 (12)
28	1,3-ditertarybutylbenzene	30.75	0.941	1418	86	**175**	175 (100), 57 (34), 190 (15), 176 (14)
29	linalool oxide	31.04	0.950	1426	88	**59**	59 (100), 94 (79), 93 (74), 43 (54), 67 (52), 55 (52), 111 (51), 68 (47), 81 (32), 79 (31)
30	benzaldehyde	33.71	1.031	1507	89	**106**	105 (100), 77 (89), 106 (87), 51 (34), 50 (21), 78 (16)
31	L-linalool	33.88	1.036	1512	86	**71**	71 (100), 93 (91), 55 (78), 43 (69), 41 (66), 69 (62), 80 (58), 121 (42), 67 (39), 92 (34)
32	1-octanol	34.21	1.046	1522	85	**56**	56 (100), 55 (99), 41 (75), 69 (68), 70 (63), 43 (52), 83 (50), 42 (46), 84 (41), 57 (36)
33	lilac aldehyde A	34.32	1.050	1526	96	**55**	55 (100), 43 (67), 93 (62), 67 (52), 111 (44), 71 (42), 41 (40), 69 (32), 81 (32), 110 (31)
34	lilac aldehyde B	34.84	1.066	1542	94	**55**	55 (100), 93 (68), 43 (61), 67 (54), 71 (53), 111 (43), 41 (42), 69 (36), 81 (30), 68 (28)
35	lilac aldehyde C	35.06	1.072	1549	96	**55**	55 (100), 93 (64), 43 (53), 67 (48), 111 (43), 41 (38), 71 (35), 69 (31), 81 (29), 153 (29)
36	hotrienol	35.80	1.095	1573	88	**82**	71 (100), 82 (67), 43 (43), 67 (39), 41 (14), 55 (11)
37	lilac aldehyde D	35.88	1.098	1575	89	**93**	55 (100), 93 (54), 43 (49), 71 (47), 67 (42), 41 (35), 111 (31), 69 (29), 153 (26), 68 (23)
38	*β*-cyclocitral	37.20	1.138	1618	86	**137**	137 (100), 152 (89), 109 (86), 81 (77), 123 (77), 67 (76), 41 (50), 91 (48), 79 (46), 43 (46)
39	phenylacetaldehyde	37.36	1.143	1623	85	**91**	91 (100), 55 (34), 56 (34), 70 (29), 41 (28), 69 (25), 92 (25), 43 (23), 65 (19), 120 (18)
40	2-methylbutrate	37.48	1.147	1627	85	**57**	74 (100), 91 (87), 57 (42), 41 (41), 87 (26), 92 (26), 73 (21), 65 (19), 39 (18), 120 (17)
41	*p*-methylbenzaldehyde	37.76	1.155	1636	86	**119**	91 (100), 119 (84), 120 (79), 65 (26), 105 (13), 39 (12), 63 (11), 89 (11), 92 (11), 77 (10)
42	*Z*-3-hexenyl angelate	38.13	1.166	1649	91	**67**	67 (100), 82 (92), 83 (87), 55 (71), 41 (19), 39 (17), 81 (11), 53 (11), 101 (10)
43	salicylic aldehyde	38.58	1.180	1664	89	**122**	122 (100), 121 (94), 65 (31), 93 (25), 45 (23), 39 (20), 76 (18), 104 (12), 40 (11), 91 (11)
44	4-oxoisophorone	39.05	1.195	1679	86	**68**	68 (100), 96 (80), 152 (33), 39 (27), 40 (26), 109 (15), 41 (13)
45	lilac alcohol A	39.87	1.220	1707	89	**93**	93 (100), 111 (98), 55 (74), 67 (50), 43 (49), 71 (30), 41 (29), 81 (29), 69 (24), 91 (24)
46	epoxylinalool	39.98	1.223	1710	87	**67**	93 (100), 111 (98), 55 (74), 67 (50), 43 (49), 71 (30), 41 (29), 81 (29), 69 (24), 91 (24)
47	lilac alcohol B	40.51	1.239	1729	94	**55**	93 (100), 111 (86), 55 (77), 43 (41), 41 (40), 67 (40), 91 (31), 69 (28), 81 (27), 71 (26)
48	lilac alcohol C	41.49	1.269	1763	93	**55**	93 (100), 111 (92), 55 (77), 67 (53), 43 (49), 41 (32), 69 (28), 81 (28), 71 (27), 91 (23)
49	methyl salicylate	41.56	1.271	1766	87	**120**	120 (100), 92 (61), 152 (47), 121 (27), 65 (17), 93 (15), 39 (13), 64 (12), 63 (11)
50	*β*-phenethyl acetate	42.37	1.296	1794	87	**104**	104 (100), 43 (30), 91 (19), 125 (18), 126 (15), 105 (10)
51	lilac alcohol D	42.55	1.302	1800	86	**55**	93 (100), 111 (99), 55 (91), 67 (51), 120 (48), 43 (46), 81 (31), 41 (31), 71 (29), 92 (27)
52	geraniol	42.74	1.307	1807	89	**123**	69 (100), 41 (59), 67 (22), 68 (21), 93 (19), 45 (16), 39 (13), 43 (12), 55 (12), 123 (11)
53	benzenemethanol	43.71	1.337	1843	98	**79**	79 (100), 108 (76), 77 (59), 107 (56), 51 (19), 91 (14), 78 (13)
54	benzeneethanol	44.79	1.370	1882	94	**91**	91 (100), 92 (62), 122 (32), 65 (22)
55	benzyl nitrile	45.41	1.389	1905	96	**117**	117 (100), 90 (61), 116 (46), 89 (43), 91 (14), 63 (14), 51 (12)
56	2-phenylbut-2-enal	45.62	1.396	1913	85	**117**	115 (100), 117 (81), 146 (56), 91 (40), 116 (34), 118 (15), 89 (14), 65 (12), 78 (12), 145 (11)

57	*β*-Ionone	46.14	1.411	1933	85	**177**	45 (100), 177 (98), 43 (53), 89 (38), 59 (30), 87 (27), 73 (27), 44 (24), 91 (23), 41 (19)
58	2-methylphenol	46.75	1.430	1956	89	**85**	68 (100), 67 (80), 85 (69), 43 (59), 83 (53), 55 (39), 71 (39), 41 (37), 82 (34), 45 (33)
59	*E*-nerolidol	47.92	1.466	2001	92	**69**	69 (100), 93 (67), 41 (59), 43 (51), 107 (47), 71 (41), 81 (33), 67 (30), 79 (27), 55 (25)
60	dimethyl salicylate	48.83	1.494	2037	91	**135**	135 (100), 77 (53), 133 (52), 45 (42), 166 (25), 92 (24), 105 (19), 89 (16), 137 (14), 63 (13)
61	ethyl linalool	50.58	1.547	2112	93	**71**	71 (100), 43 (77), 45 (46), 55 (45), 87 (41), 83 (36), 41 (34), 82 (28), 69 (26), 67 (23)
62	eugenol	51.23	1.567	2154	93	**164**	164 (100), 45 (64), 103 (44), 149 (40), 77 (36), 131 (36), 91 (34), 133 (32), 89 (31), 121 (23)
63	8-hydroxy-6,7-dihydrolinalool	51.62	1.579	2180	91	**71**	71 (100), 43 (36), 45 (34), 55 (20), 89 (15), 41 (14), 59 (12), 69 (11), 72 (11)
64	methyl palmitate	52.69	1.612	2267	86	**74**	74 (100), 87 (64), 43 (25), 55 (23), 75 (19), 143 (17), 41 (15), 69 (15), 57 (14)
65	2-hydroxylinalool	53.14	1.626	2307	85	**43**	43 (100), 71 (86), 67 (79), 45 (60), 55 (40), 41 (34), 68 (30), 89 (26), 119 (25), 82 (24)
66	1,3-diacetylbenzene	55.68	1.703	2496	85	**147**	147 (100), 91 (41), 119 (21), 162 (16), 43 (16), 76 (11), 148 (10)
ID[g]	1,2-dichlorobenzene-d_4	32.69	1.000	1476	98	**150**	150 (100), 152 (88), 115 (75), 78 (45), 117 (28), 52 (25), 154 (25), 151 (24), 76 (22), 75 (13)

[a]Retention time (min); [b]Relative retention time, retention time of analyte/retention time of 1,2-dichlorobenzene-d_4); [c]Linear retention indices calculated against *n*-alkanes (C_7-C_{30}); [d]Nist library match values (100 = 100% identical to the NIST library entry); [e]quantification ion (*m*/*z*), Specific mass ion used for quantification; [f]Lists of the representative ion peaks which intensity relatively more than 10% of biggest peak; [g]Iintenal strandard.

Table S2. Classification and odor note of the detected volatile components identified from the flowers in five variants of *Abeliophyllum distichum* using HS-SPME-GC/MS analysis

No	Compound	Retention time	Classification	Odor note
1	linalool oxide	31.04	Oxygenated monoterpenes	flower [a]
2	L-linalool	33.88	Oxygenated monoterpenes	lemon, orange, citrus, floral, sweet, bois de rose, woody, green, blueberry [b]
3	lilac aldehyde A	34.32	Oxygenated monoterpenes	flowery, fresh [b]
4	lilac aldehyde B	34.84	Oxygenated monoterpenes	flowery, fresh [b]
5	lilac aldehyde C	35.06	Oxygenated monoterpenes	sweet, flowery [b]
6	hotrienol	35.80	Oxygenated monoterpenes	sweet, tropical, floral, fennel, ginger, spicy [b]
7	lilac aldehyde D	35.88	Oxygenated monoterpenes	sweet, flowery [b]
8	*β*-cyclocitral	37.20	Oxygenated monoterpenes	tropical, saffron, herbal, clean, rose, sweet, tobacco, green, fruity [b]
9	lilac alcohol A	39.87	Oxygenated monoterpenes	flowery, fresh [b]
10	epoxylinalool	39.98	Oxygenated monoterpenes	flower [a]
11	lilac alcohol B	40.51	Oxygenated monoterpenes	flowery, fresh [b]
12	lilac alcohol C	41.49	Oxygenated monoterpenes	flowery, fresh [b]
13	lilac alcohol D	42.55	Oxygenated monoterpenes	flowery, fresh [b]
14	ethyl linalool	50.58	Oxygenated monoterpenes	fresh, bois de rose, herbal, wet, green, lavender, bergamot [b]
15	8-hydroxy-6,7-dihydrolinalool	51.62	Oxygenated monoterpenes	bois de rose, woody, citrus, blueberry, weedy [b]
16	2-hydroxylinalool	53.14	Oxygenated monoterpenes	green, minty, spicy, herbal, rosemary, phenolic [b]
17	*E*-nerolidol	47.92	Sesquiterpenes alcohols	wood, flower, wax [a]
18	*β*-myrcene	20.94	Monoterpene hydrocarbons	balsamic, must, spice [a]
19	limonene	22.62	Monoterpene hydrocarbons	citrus, mint [a]
20	ocimene isomer-1	23.54	Monoterpene hydrocarbons	citrus, herb, flower [a]
21	ocimene isomer-2	24.21	Monoterpene hydrocarbons	citrus, herb, flower [a]
22	allo-ocimene	28.60	Monoterpene hydrocarbons	fruit, wet cloth [a]
23	benzaldehyde	33.71	Aromatic alcohols	fruity [b]
24	phenylacetaldehyde	37.36	Aromatic alcohols	honey, flower [a]
25	*p*-methylbenzaldehyde	37.76	Aromatic alcohols	fruity, cherry, phenolic [b]
26	salicylic aldehyde	38.58	Aromatic alcohols	medicinal, spicy, cinnamon, wintergreen, cooling [b]
27	methyl salicylate	41.56	Aromatic alcohols	peppermint [a]
28	*β*-phenethyl acetate	42.37	Aromatic alcohols	rose, honey, tobacco [a]
29	geraniol	42.74	Aromatic alcohols	rose, geranium [a]
30	benzenemethanol	43.71	Aromatic alcohols	sweet, floral, fruity [b]
31	benzeneethanol	44.79	Aromatic alcohols	floral, rose, dried rose [b]
32	2-phenylbut-2-enal	45.62	Aromatic alcohols	sweet, narcissus, cortex, beany, honey, cocoa, nutty, radish [b]
33	dimethyl salicylate	48.83	Aromatic alcohols	wintergreen, mint [a]
34	eugenol	51.23	Aromatic alcohols	clove, honey [a]
35	*p*-cymene	28.65	Aromatics	solvent, gasoline, citrus [a]
36	1,3-ditertarybutylbenzene	30.75	Aromatics	-
37	benzyl nitrile	45.41	Aromatics	-
38	2-methylphenol	46.75	Aromatics	musty, phenolic, plastic, medicinal, herbal, leathery [b]
39	1,3-diacetylbenzene	55.68	Aromatics	-
40	ethanol	11.91	Alcohols	sweet [a]
41	2-butyl-3-octanol	19.22	Alcohols	-
42	isoamylalcohol	21.98	Alcohols	pungent [b]
43	*n*-hexyl acetate	24.85	Alcohols	fruit, herb [a]
44	isohexanol	25.95	Alcohols	resin, flower, green [a]
45	3-methyl-1-pentanol	26.43	Alcohols	pungent [a]
46	2-hexenyl acetate	26.92	Alcohols	green, banana [a]
47	1-hexanol	27.32	Alcohols	resin, flower, green [a]
48	2-methyl-1-decanol	27.80	Alcohols	-
49	*Z*-3-hexenol	28.47	Alcohols	green, cortex, privet, leafy, floral, petal, oily, earthy [b]
50	2-hexenol	29.14	Alcohols	leaf, green, wine, fruit [a]
51	1-octen-3-ol	30.61	Alcohols	soap, plastic [a]
52	1-octanol	34.21	Alcohols	mushroom, fat [a]
53	isovaleraldehyde	11.50	Aldehyde & Carboxyls	ethereal, aldehydic, chocolate, peach, fatty [b]
54	*n*-hexanal	17.67	Aldehyde & Carboxyls	grass, tallow, fat [a]
55	2-hexenal	22.91	Aldehyde & Carboxyls	apple, green [a]
56	6-methyl-5-hepten-2-one	27.19	Aldehyde & Carboxyls	-
57	nonaldehyde	29.39	Aldehyde & Carboxyls	waxy, aldehydic, rose, fresh, orris, orange peel, fatty [b]
58	acetic acid	30.42	Aldehyde & Carboxyls	sour [a]
59	2-methylbutrate	37.48	Aldehyde & Carboxyls	pungent, acidic, cheesy, roquefort, cheese [b]

60	*Z*-3-hexenyl angelate	38.13	Aldehyde & Carboxyls	green, floral, gardenia, jasmin [b]
61	4-oxoisophorone	39.05	Aldehyde & Carboxyls	musty, woody, sweet, tea, citrus, lemon, brown [b]
62	*β*-lonone	46.14	Aldehyde & Carboxyls	seaweed, violet, flower, raspberry [b]
63	decane	15.26	Others	-
64	5-ethyl-2,2,3-trimethylheptane	16.82	Others	-
65	*E*-3-eicosene	27.49	Others	-
66	methyl palmitate	52.69	Others	oily, waxy, fatty, orris [b]

[a]Scent database of Flavornet [1]; [b]Scent database of The good scents company [2]

Reference

1. Flavornet and human odor space. Available online: http://www.flavornet.org/flavornet.html (accessed on 12 November 2019).
2. The good scents company. Available online: http://www.thegoodscentscompany.com (accessed on 12 November 2019).

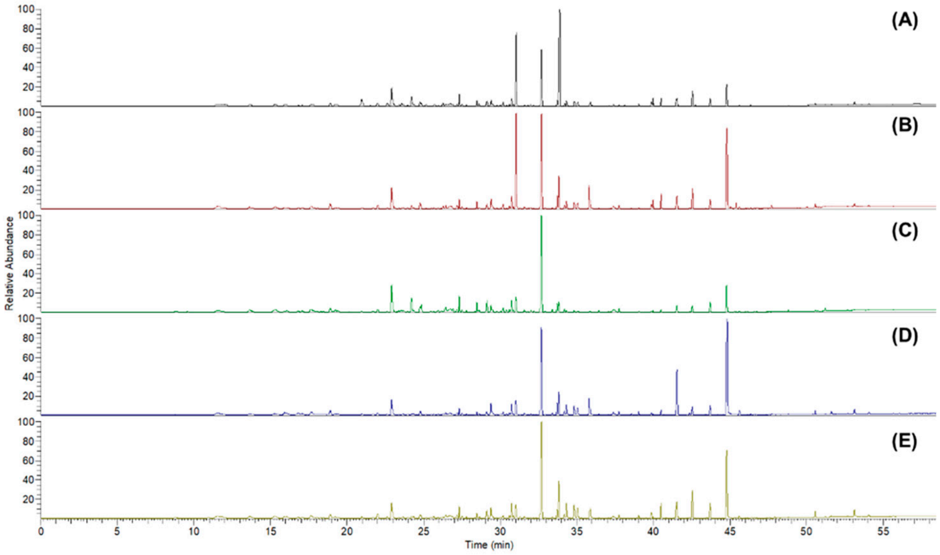

Figure S1. Representative total ion chromatograms (TIC) for fresh tissue of five variants of *Abeliophyllum distichum* flowers. (A) White miseon, (B) pink miseon, (C) ivory miseon, (D) blue miseon, (E) round miseon.

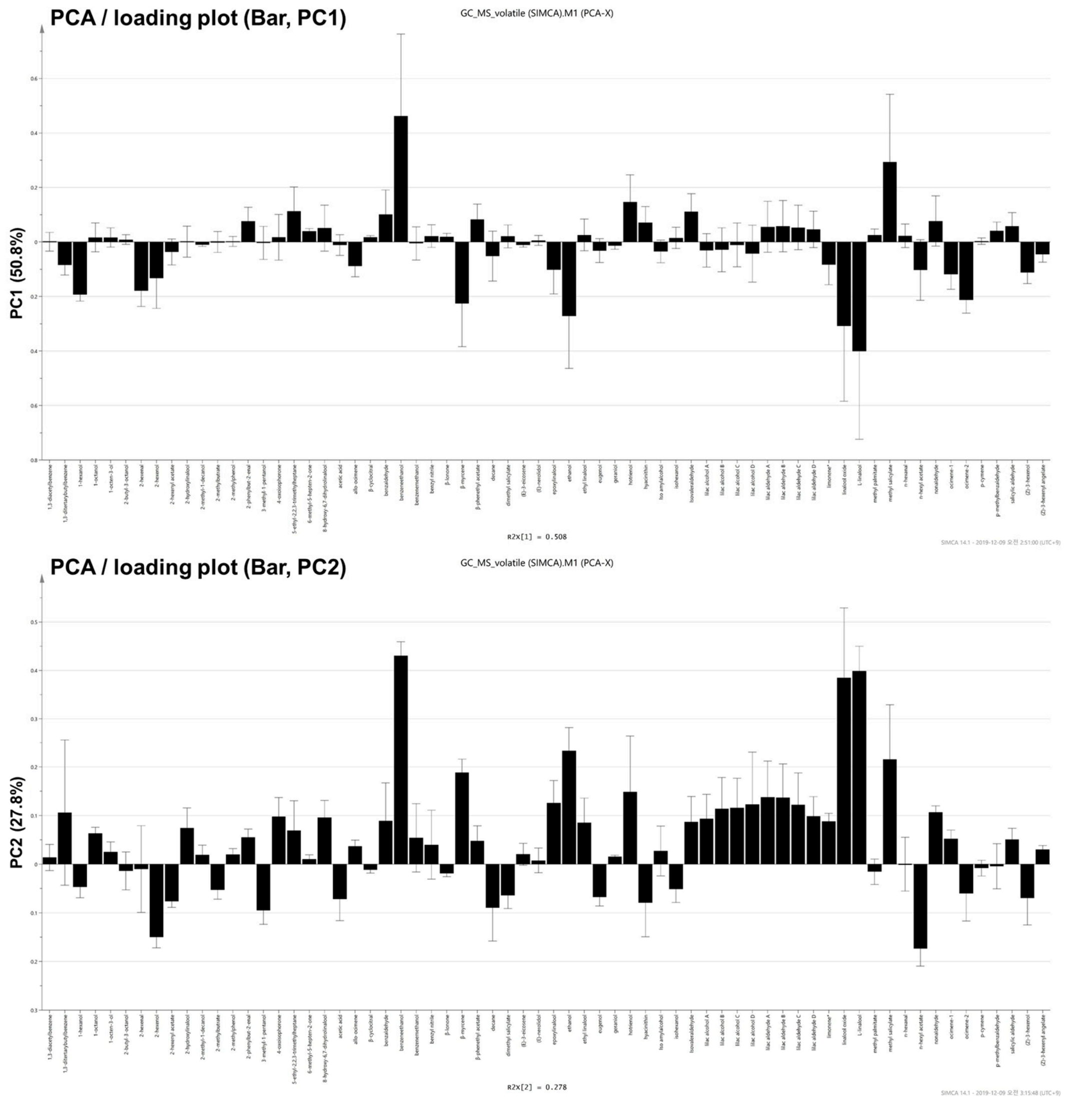

Figure S2. PCA score plot obtained from HS-SPME-GC/MS results on five variants of *Abeliophyllum distichum* flowers.

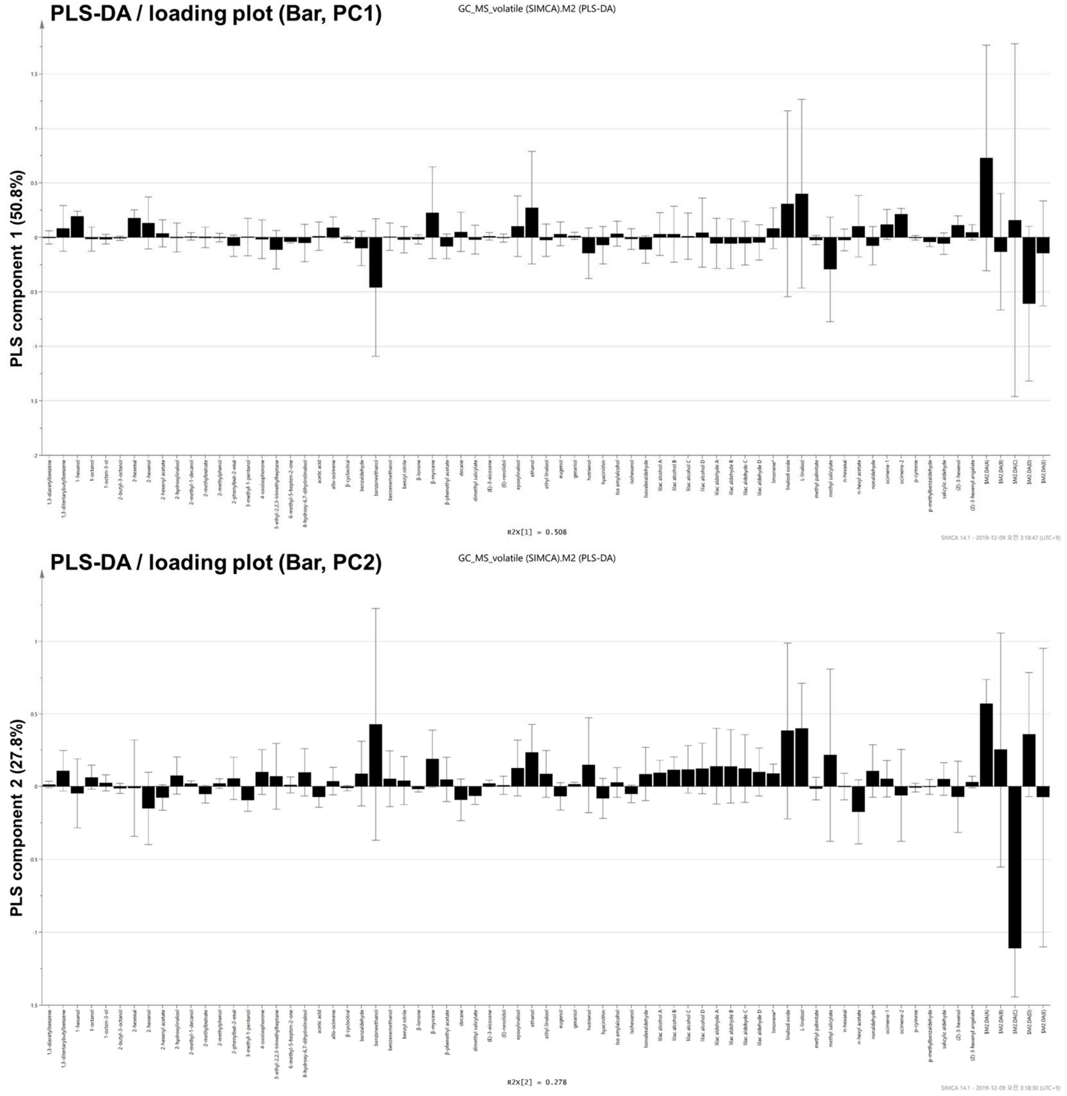

Figure S3. PLS-DA score plot obtained from HS-SPME-GC/MS results on five variants of *Abeliophyllum distichum* flowers.
